# Lymphoid and myeloid immune cell reconstitution after nicotinamide-expanded cord blood transplantation

**DOI:** 10.1038/s41409-021-01417-4

**Published:** 2021-07-26

**Authors:** Coco de Koning, Weiyang Tao, Amelia Lacna, Karin van Veghel, Mitchell E. Horwitz, Guillermo Sanz, Madan H. Jagasia, John E. Wagner, Patrick J. Stiff, Rabi Hanna, Daniela Cilloni, David Valcárcel, Tony Peled, Einat Galamidi Cohen, Uri Goshen, Aridaman Pandit, Caroline A. Lindemans, Jaap Jan Boelens, Stefan Nierkens

**Affiliations:** 1 University Medical Center Utrecht, Utrecht, The Netherlands; 2 Princess Máxima Center for Pediatric Oncology, Utrecht, The Netherlands; 3 Duke University Medical Center, Durham, NC USA; 4 Hospital Universitario y Politécnico la Fe, València, Spain; 5 Centro de Investigación Biomédica en Red de Cáncer, CIBERONC, Instituto de Salud Carlos III, Madrid, Spain; 6 Vanderbilt University Medical Center, Nashville, TN USA; 7 University of Minnesota, Minneapolis, MN USA; 8 Loyola University Medical Center, Chicago, IL USA; 9 Cleveland Clinic, Cleveland, OH USA; 10 University of Turin, Turin, Italy; 11 University Hospital Vall d’Hebron, Barcelona, Spain; 12 Gamida Cell. Ltd, Jerusalem, Israel; 13 Memorial Sloan Kettering Cancer Center, New York, NY USA

**Keywords:** Medical research, Immunology

## Abstract

Omidubicel (nicotinamide-expanded cord blood) is a potential alternative source for allogeneic hematopoietic cell transplantation (HCT) when an HLA-identical donor is lacking. A phase I/II trial with standalone omidubicel HCT showed rapid and robust neutrophil and platelet engraftment. In this study, we evaluated the immune reconstitution (IR) of patients receiving omidubicel grafts during the first 6 months post-transplant, as IR is critical for favorable outcomes of the procedure. Data was collected from the omidubicel phase I-II international, multicenter trial. The primary endpoint was the probability of achieving adequate CD4+ T-cell IR (CD4IR: > 50 × 10^6^/L within 100 days). Secondary endpoints were the recovery of T-cells, natural killer (NK)-cells, B-cells, dendritic cells (DC), and monocytes as determined with multicolor flow cytometry. LOESS-regression curves and cumulative incidence plots were used for data description. Thirty-six omidubicel recipients (median 44; 13–63 years) were included, and IR data was available from 28 recipients. Of these patients, 90% achieved adequate CD4IR. Overall, IR was complete and consisted of T-cell, monocyte, DC, and notably fast NK- and B-cell reconstitution, compared to conventional grafts. Our data show that transplantation of adolescent and adult patients with omidubicel results in full and broad IR, which is comparable with IR after HCT with conventional graft sources.

## Introduction

Immune reconstitution (IR) is an important predictor for outcome after allogeneic hematopoietic (stem) cell transplantation (HCT) [1–8]. IR can be affected by a variety of factors, such as conditioning regimen and cell source [9, 10]. Common first-choice sources for allogeneic HCT in adults are peripheral blood stem cells (PBSC) or bone marrow (BM) from related or unrelated donors. Nevertheless, for many adult patients, no PBSC or BM donor can be found, because of the requirement for high-grade human leukocyte antigen (HLA)-matching. The use of umbilical cord blood (CB) provides an alternative cell source in these patients since lower-grade HLA-matching has proven to be acceptable to ensure a low risk of graft failure or graft-versus-host disease (GvHD) [11–13]. However, in order to ensure timely engraftment, patients must receive an adequate dose of stem cells/kg [14, 15]. This may introduce a problem when using CB as a stem cell source in adults, since CB-grafts generally contain lower amounts of nucleated (stem) cells compared to PBSC/BM, resulting in delayed engraftment. One option to overcome the limited amount of stem cells is transplantation using two CB-grafts (double cord blood transplantation; dUCBT) [16, 17]. However, dUCBT still does not overcome the delayed engraftment and may be associated with an increased risk of GvHD [18–20]. Methods to expand CB stem cells provide another option to enhance CB-graft availability for adult patients. One option to expand CB stem cells is a nicotinamide-based protocol: omidubicel (Gamida Cell, Jerusalem, Israel).

Omidubicel is an ex vivo expanded cell product derived from the CD133+ fraction of banked CB that uses an epigenetic strategy to inhibit differentiation and enhances the functionality of cultured hematopoietic stem and -progenitor cells. Nicotinamide is the active agent of this expansion strategy. When nicotinamide is added to stimulatory hematopoietic cytokines, CB-derived hematopoietic progenitor cell cultures demonstrate an increased frequency of phenotypically primitive CD34+ CD38− cells and a substantial increase in BM homing and engraftment potential of ex vivo expanded CD34+ cells [21–26]. Omidubicel is comprised of ex vivo expanded stem cell fraction (omidubicel cultured fraction (CF)) and a non-cultured cell fraction of the same CB unit (omidubicel non-cultured fraction (NF)) consisting of mature myeloid and lymphoid cells. A phase I/II trial with standalone omidubicel HCT showed rapid neutrophil (11.5 days) and platelet engraftment (34 days) [23]. This early hematopoietic recovery reflects the ability of nicotinamide to expand both committed and long-term repopulating hematopoietic stem cells and was shown to be associated with a lower risk of bacterial infections and shorter hospitalization in the first 100 days compared with standard unmanipulated CB transplantation (unCBT) [22, 23].

We and others have recently shown that adequate CD4+ T-cell IR (CD4 + IR) is crucial for favorable survival outcomes [1, 2, 6–8]. Patients with adequate CD4 + IR (>50 × 10^6^ CD4+ T-cells/L blood, within 100 days after HCT) had a lower risk of viral reactivation [1], virus-related morbidity and mortality [1], aGvHD-related mortality [27], and relapse-related mortality [6, 8]. In addition, IR of other immune cell subsets, such as natural killer (NK)-cells [28–30], CD8+ T-cells [31–33], and B-cells [34–39], were also related to HCT outcome. As a result of the manufacturing process manipulations and freeze-thaw cycles, the CD3+ dose of the omidubicel NF is lower than in a standard CBT. For example, Purtill et al. reported a median infused viable dose 4.27 × 10^6^ CD3+ cells/kg [40]. This is also consistent with the previously published omidubicel experience, indicating that the median CD3+ cell dose from the omidubicel unit was 1.3 × 10^6^ cells/kg, which was significantly lower than the median cell dose from the unmanipulated unit of 3.4 × 10^6^ cells/kg [41]. The omidubicel CF yields hematopoietic stem and myeloid progenitor cells, but lymphoid cells cannot be detected based on cell surface marker analysis. However, the development of lymphoid cell subsets occurs de-novo from the expanded CD34+ stem and progenitor cells post-transplant [21, 23, 24]. The NF contains mature lymphocytes. The lower CD3+ dose in omidubicel may theoretically be postulated to contribute to a risk of impaired immune recovery following transplantation. Nevertheless, detailed information on IR after transplantation with omidubicel has not yet been reported to date.

We performed in-depth immune monitoring and evaluated plasma protein profiles in patients transplanted with omidubicel grafts in a phase I/II international multicenter study. For this, we developed multicolor flow cytometry panels for harmonized measurements to evaluate the recovery of T-, B-, natural killer (NK)-cell, monocyte, and dendritic cell (DC) subsets.

## Patients and methods

### Patients and treatment

In this phase, I/II multicenter trial, patients with hematologic malignancies received an omidubicel-HCT after myeloablative (MA) conditioning without antithymocyte globulin (ATG), at 11 clinical sites throughout the United States, Europe, and Singapore. Conditioning regimens were applied according to local standard protocols, and are described in a previous publication of this trial [23]. The study was approved by the institutional review boards of all participating institutions and the national regulatory authorities. All patients provided written informed consent. Patients were enrolled, and data were collected and registered prospectively only after written informed consent. The study was performed in accordance with the International Conference on Harmonization Guidelines and Good Clinical Practice (ClinicalTrials.gov identifier: NCT01816230).

### Immune monitoring and blood cell samples

Immune monitoring was performed on peripheral blood samples drawn at 7, 14, 21, 42, 70, and 180 days after infusion of the graft. Absolute leukocyte, lymphocyte, neutrophil, and monocyte cell numbers were measured in fresh EDTA-whole blood. Mononuclear cells were isolated using Fico-II-Paque (BD Biosciences, Sweden) and cryopreserved for later measurements with the use of standardized and validated multicolor flow cytometry panels. The subsets identified were; for T-cells: naïve (CCR7+ CD27+ CD45RO− CD45RA+), central memory (CCR7+ CD27+ CD45RO+ CD45RA−), effector memory (CCR7− CD27− CD45RO+ CD45RA−), Temra (CCR7− CD27− CD45RO− CD45RA+), and Treg (CD4+ CD25+ CD127^low^FoxP3+) [42, 43]. The T helper cell phenotypes were based on chemokine receptor expression shown to be associated with Th helper subsets and in this manuscript are referred to as “Th1” (CD4+ CXCR3+ CCR4− CCR6−), “Th2” (CD4+ CCR6− CXCR3− CCR4+), “Th17” (CD4+ CCR6+ CXCR3+ CCR4− CCR10−), “Th22” (CD4+ CCR6+ CXCR3− CCR4+ CCR10+) [42, 43]. B-cells were characterized into immature (CD19+ CD24++ CD38++ IgM+ IgD−), transitional (CD19+ CD24++ CD38++ IgM+ IgD+), follicular (CD19+CD24+CD38+IgM+IgD+), memory (CD19+ CD24− CD38+), and plasmablast (CD19+ CD24− CD28++) [44, 45]. For other subsets, markers were as follows: for NK-cells: naïve (CD3− CD56++ CD16−) and effector (CD3− CD56+ CD16+; for NK T-cells: NKT (CD3+ CD56+)), invariant NKT (iNKT; CD3+ CD56+ TCRVbeta11+ TCRValpha24+) [46, 47]; for monocytes: classical (lin-HLA.DR+ CD14+ CD16−), intermediate (lin-HLA.DR+ CD14+ CD16+), non-classical (lin-HLA.DR+ CD14− CD16+) [48]; and for DCs: conventional (cDC; lin-HLA.DR + CD14-CD16-CD11c + CD123−; available numbers of cells were too low to report DC1 versus DC2) and plasmacytoid (pDC; lin-HLA.DR+ CD14− CD16− CD11c− CD123+) [49, 50]. An overview of the monoclonals used is provided in Supplemental Table 1. Subsets were calculated as the percentage of total evaluable immune cells. Absolute lymphocyte, leukocyte, and monocyte counts were available from standard immune monitoring on fresh material. Absolute numbers of immune cell subsets from in-depth immune monitoring were calculated from total absolute lymphocyte number (for T-, B-, NK-cells) or leukocyte number (for DC).

### Luminex and plasma samples

Plasma was collected by centrifuging EDTA-whole blood samples, from the same samples as for immune monitoring on blood cells, at 0, 1, 7, 14, 21, 42, and 70 days after transplantation. A total of 60 plasma proteins were measured per sample using multiplex immunoassays (Luminex Technology); IL1RA, IL2, IL3, IL4, IL5, IL6, IL7, IL10, IL15, IL17, IL18, IL22, TNFα, IFNα, IFNγ, APRIL, OSM, LAG3, Follistatin, I309, MIP1a, MIP1b, IL8, MIG, IP10, BLC, OPG, OPN, G-CSF, M-CSF, GM-CSF, SCF, HGF, EGF, AR, VEGF, CD40L, sPD1, FASL, IL1R1, IL1R2, ST2, TNFR1, TNFR2, sIL2Rα, sCD27, IL7Rα, sSCFR, Elastase, S100A8, Gal9, Ang1, Ang2, LAP, TPO, sICAM, sVCAM, MMP3, Gal3, C5a. The multiplex immunoassay was performed according to the protocol from the MultiPlex Core Facility of the UMCU [51].

### Data analysis

The primary endpoint was the probability of achieving CD4 + IR; > 50 × 10^6^ CD3+ CD4+ cells/L blood on two consecutive measurements within 100 days. Secondary endpoints were IR over time of CD3+, CD8+, and CD4+ T-cell subsets, monocytes, NK- and B-cell subsets during 0–180 days after HCT. LOESS-regression curves and cumulative incidence plots were used for data description. The relation between plasma protein- and IR data was analyzed using Spearman regression. R software (version 4.0.1) and ggplot2 package (version 3.3.3) were used for data analysis and production of graphs [52].

## Results

In this phase I/II multicenter trial, 36 omidubicel recipients (median age 44; range 13–63 years) were included. Patient characteristics are described elsewhere [23]. Omidubicel cell dose consisted of a median 6.3 × 10^6^ CD34+/kg, and 2.4 × 10^6^ CD3+ T-cells/kg of the co-infused negative fraction (following CD133+ selection). Informed consents for in-depth immune monitoring were available from 28 omidubicel recipients. There was no selection bias for these 28 consented patients, based on median age (39 years [13–63]) and median cell dosages of 6.1 × 10^6^ CD34+/kg and 2.4 × 10^6^ CD3+T-cells/kg.

### IR after omidubicel transplantation

**Fig. 1:**
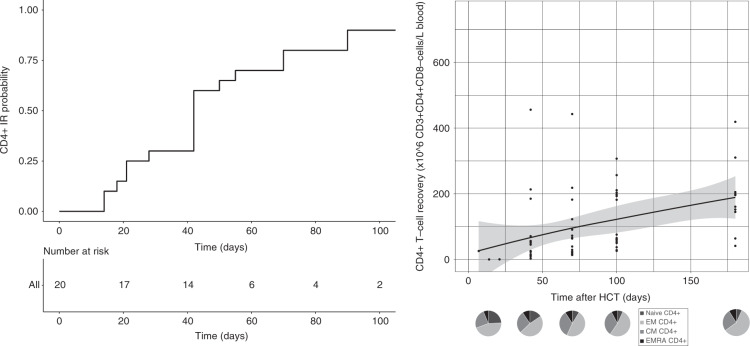
CD4+T-cell reconstitution after omidubicel transplantation. (Left) Cumulative incidence curve of CD4+IR probability after transplantation with omidubicel transplantation. CD4+ IR was evaluable in 20 patients and was defined as having >50*10^6^ CD4+ T-cells/L in two consecutive measurements within 100 days after transplantation. (Right) Smoothened LOESS-curve with 95% confidence interval (gray area), with dots showing the data points, for absolute CD4+ T-cell counts following omidubicel transplantation. Each dot represents a single data point for a single patient. (Lower, right) Pie-charts of CD4+ T-cell subsets as percentages; naïve, effector memory (EM), central memory (CM), and EMRA T-cells, at 7–14, 21, 42, 70, and 180 days after transplantation.

**Fig. 2:**
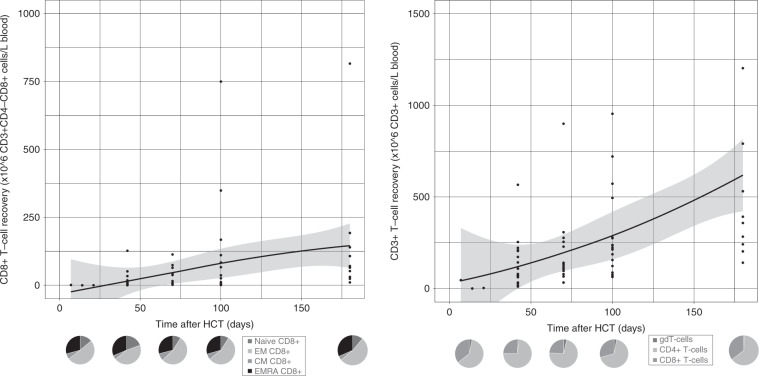
CD8+and total T-cell reconstitution after omidubicel transplantation. (Upper) Smoothened LOESS-curve with 95% confidence interval (gray area), with dots showing the data points, for absolute CD8+ (left) and total (right) T-cell counts following omidubicel transplantation. Each dot represents a single data point for a single patient. (Lower) Pie-charts of CD8+T-cell subsets as percentages; naïve, effector memory (EM), central memory (CM) and EMRA T-cells, and total T-cell subsets as percentages; gamma-delta T-cells, CD4+ and CD8+ T-cells, at 7–14, 21, 42, 70, and 180 days after transplantation.

**Fig. 3:**
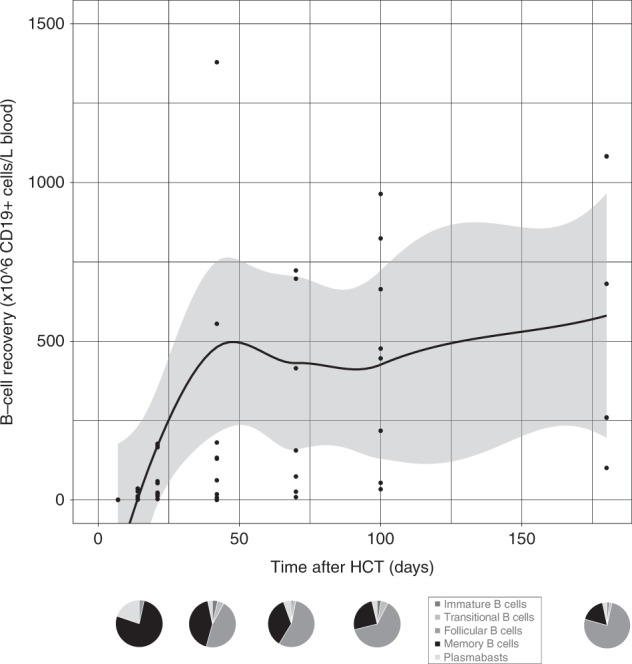
B-cell reconstitution after omidubicel transplantation. (Upper) Smoothened LOESS-curve with 95% confidence interval (gray area), with dots showing the data points, for absolute B-cell counts following omidubicel transplantation. Each dot represents a single data point for a single patient. (Lower) Pie-charts of B-cell subsets as percentages of B-cells; immature, transitional, follicular, memory B-cells, and plasma cells, at 7–14, 21, 42, 70, and 180 days after transplantation.

**Fig. 4:**
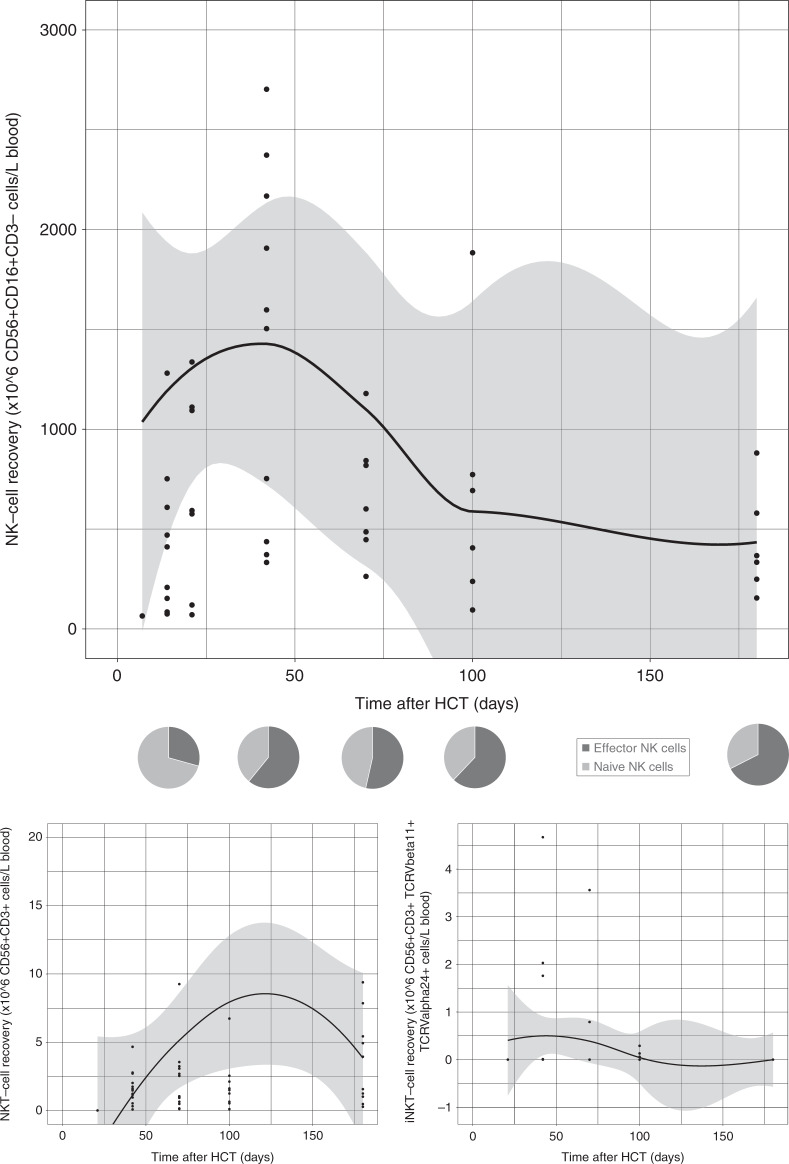
NK- and NKT-cell reconstitution after omidubicel transplantation. (Upper) Smoothened LOESS-curve with 95% confidence interval (gray area), with dots showing the data points, for absolute NK-cell counts following omidubicel transplantation. Each dot represents a single data point for a single patient. (Middle) Pie-charts of NK-cell subsets as percentages of NK-cells; effector and naïve NK-cells, at 7–14, 21, 42, 70, and 180 days after transplantation. (Lower) Smoothened LOESS-curves of NKT- and iNKT-cell recovery after omidubicel transplantation.

**Fig. 5:**
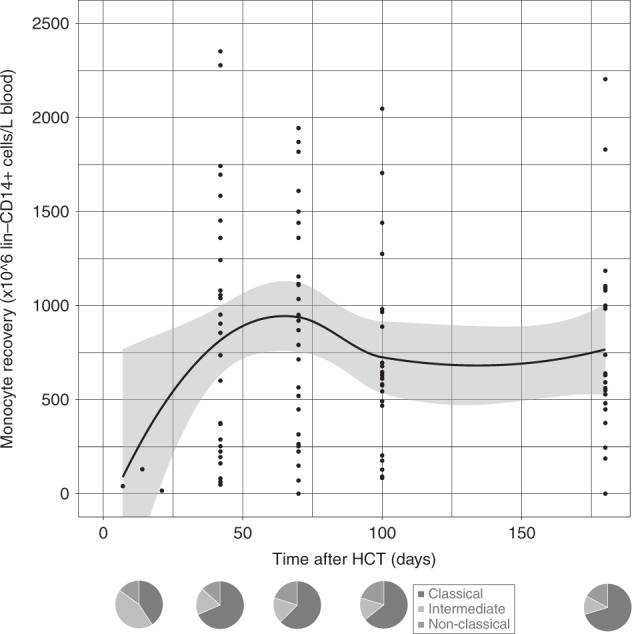
Monocyte reconstitution after omidubicel transplantation. (Upper) Smoothened LOESS-curve with 95% confidence interval (gray area), with dots showing the data points, for absolute monocyte counts following omidubicel transplantation. Each dot represents a single data point for a single patient. (Lower) Pie-charts of monocyte subsets as percentages of total monocytes; classical, intermediate, and non-classical monocytes, at 7–14, 21, 42, 70, and 180 days after transplantation.

**Fig. 6:**
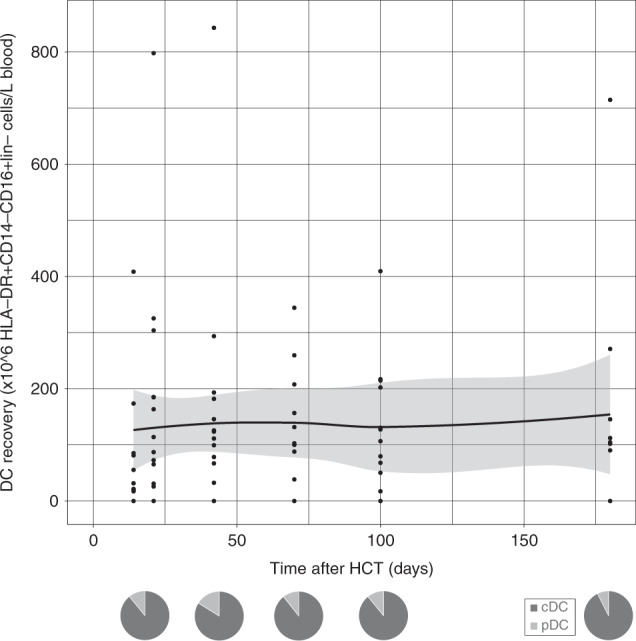
Dendritic cell reconstitution after omidubicel transplantation. (Upper) Smoothened LOESS-curve with 95% confidence interval (gray area), with dots showing the data points, for absolute DC counts following omidubicel transplantation. Each dot represents a single data point for a single patient. (Lower) Pie-charts of DC subsets as percentages of total DCs; conventional (cDC) and plasmacytoid DCs (pDC), at 7–14, 21, 42, 70, and 180 days after transplantation.

Since early adequate CD4+ IR has been related to lower infection-related morbidity and lower overall mortality [1–3, 7, 53], we evaluated CD4+ IR probability in omidubicel recipients. CD4+ IR was evaluable in 20 patients; not all 28 patients had data on absolute CD4+ T cell count on two consecutive time points within the first 100 days after HCT. This was either due to death prior to achieving two data points or limited lymphocyte data available to calculate absolute CD4+ T cell count. Eighteen (90%) achieved successful CD4+ IR (Fig. 1-left). Including all 28 consented patients, adaptive immune cell reconstitution of overall CD4+ (Fig. 1-right), CD8+ (Fig. 2-left), and CD3+ (Fig. 2-right) T-cells, and B-cells (Fig. 3), was observed within the first 6 months after omidubicel transplantation, with a slightly earlier recovery of innate immune cells, including NK-cells (Fig. 4), monocytes (Fig. 5), and DCs (Fig. 6). Especially, the reconstitution of B- and NK-cells was fast compared to other cell subsets, with early high cell counts of ∼500 and ∼1500 × 10^6^ cells/L blood, respectively, within the first month after transplantation.

### In-depth immune monitoring after omidubicel transplantation

The recovery of T-cell subsets during the first 6 months after omidubicel transplantation is broad and full in terms of the presence of effector and central memory CD4+ and CD8+ T-cells, gamma-delta T-cells, Tregs, Th2, Th1, and Th17 cells (Figs. 1 and 2, Supplemental Fig. 1). Within total CD3+ T-cells, relative amounts of gamma-delta T- (1–4%), CD8+ (24–36%), and CD4+ (61–74%) T-cells are normalized within the first month after transplantation. Both CD8+ and CD4+ T-cell recovery are predominately effector memory T-cells (∼50%), although relatively high naïve T-cell counts within the first month after omidubicel transplantation are observed compared to later time points (∼20% versus ∼7%). Furthermore, the second most predominant subsets are Temra (29–55%) in CD8+ T-cell recovery, and central memory T-cells (19–24%) in CD4+ reconstitution. In addition, although absolute counts of Tregs, as well as Th2, Th1, and Th17, remain low, the relative amount of Tregs in some patients (∼5% [range; 1927%] of total CD4+ T-cells) might be slightly higher than in healthy adults (2–3% of CD4+ T-cells) [54]. B-cell reconstitution starts with relatively high amounts of memory B-cells during the first weeks after transplantation, followed by increased percentages of follicular B-cells (Fig. 3). NK-cell recovery starts with relatively high naïve NK-cell counts within the first weeks, after which generally more effector NK-cells are observed (Fig. 4), with only low amounts of NKT and iNKT cells. Furthermore, monocyte recovery starts with relatively high amounts of intermediate monocytes in the first month after omidubicel transplantation, after which most monocytes are classical monocytes, with lower amounts of intermediate and non-classical monocytes (Fig. 5). DC reconstitution during the first year after omidubicel transplantation consists of primary cDCs and few pDCs (Fig. 6).

### Plasma protein profiles in relation to immune subset reconstitution

**Fig. 7:**
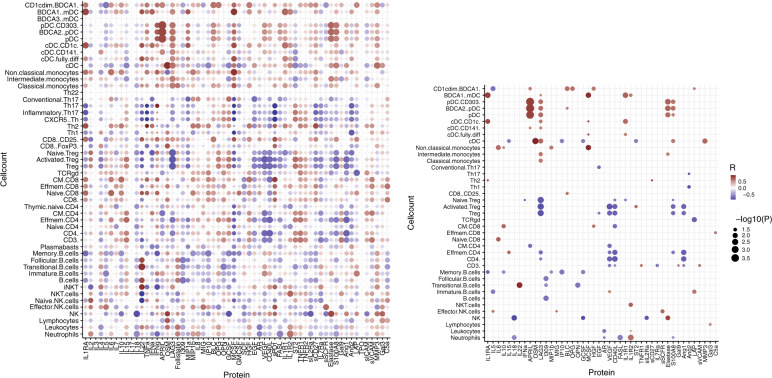
Correlation between plasma protein profiles and immune subset recovery. Overall (left) and statistically significant (*p* < 0.05; right) Spearman correlations between plasma concentrations on day 1 after transplantation and absolute immune cell subsets counts on day 21, 42, and 70 after transplantation. In the case of multiple data points for absolute immune cell counts, the mean of these data points was taken as a datapoint for immune cell count. Red colors indicate positive correlations, blue colors indicate negative correlations.

We evaluated correlations between plasma proteins at days 0, 1, and 7 with immune subset reconstitution at days 21, 42, 70, 100, and 180 in all 28 patients. The correlations between plasma proteins measured at day 1 and IR at day 21–70 were most robust, with the least missing data, and best represented the overall observations (Fig. 7). An overview of all plasma protein profiles over time after transplantation is provided in Supplemental Fig. 2.1–2.4. Interestingly, plasma IL15 concentration followed a similar recovery trend compared to the NK-cell counts, with a peak observed after one week for IL15 and around 6 weeks for NK-cells (Fig. 4 and Supplemental Fig. 2.1). In accordance with this observation, we found an, albeit weak, correlation between IL15 at day 1 and NK-cell counts at days 21, 42, and 70 (Fig. 7). Furthermore, increased IL2 concentrations correlated with counts of almost all T-cell subsets only, while increased sPD1 was correlated to increased CD8+ T-cell and to decreased CD4+ T-cell subset counts. IL22 is positively correlated with B-cell subset recovery. We further observed that ST2 was positively correlated with adaptive immune cell counts (T- and B-cell subsets), but negative correlations were found with innate immune cell recovery (DC-, monocyte, and NK-cell subsets). In turn, LAG3, CD40L, APRIL, VEGF, Elastase, S100A8, and M-CSF were positively correlated to innate immune cell recovery, and negatively with adaptive immune cell counts.

## Discussion

This unique international, multicenter, in-depth immune monitoring study reveals rapid and robust IR after transplantation with omidubicel. The probability of early CD4+ IR was high, overall CD4+ and CD8+ T-cell, monocyte, and DC reconstitution were observed, and recoveries of B-cells and NK-cells were strikingly fast after omidubicel transplantation. Together with the recently reported clinical outcome of this phase I/II study, our findings indicate that transplantation with omidubicel is not only feasible in terms of potent engraftment [23] but also results in a full and broad IR.

The fast NK- and B-cell reconstitution after omidubicel transplantation in comparison to other graft sources, might be related to an intrinsic characteristic of cord as previously described in unmanipulated-CBT recipients [10, 55, 56]. The higher number of progenitor cells obtained with omidubicel expansion may also contribute to the enhanced NK- and B-cell reconstitution, although as previously discussed, the cultured cells do not contain mature lymphocytes or NK cells, and the non-cultured cells contain a reduced number of cells compared to a standard CB unit [25, 57]. It would, therefore, be interesting to evaluate if fast NK-cell recovery after omidubicel transplantation would translate to a lower relapse risk or viral reactivation incidence as observed after unmanipulated-CBT [58, 59]. These analyses must, however, be performed in a comparative setting. In addition, improved NK-cell and NKT-cell recovery have been associated with improved overall survival and risk of infection [3, 60], as well as reduced aGvHD risk specifically for the CD56^bright^ NK-subset [28]. Also, early reconstitution of Tregs seems to protect against aGvHD development [61–63], and a Th17/Treg ratio <1 correlated favorably with aGvHD development and severity [63]. Therefore, it is of high clinical interest to further study in-depth IR, in terms of immune cell subsets, to correlate IR after omidubicel transplantation to the outcome and subsequently find possible biomarkers that can predict outcome in future omidubicel recipients. The current international multicenter phase III trial allows for further IR studies with an increased number of patients, and inclusion of a randomized control cohort of single and double CBT, and may allow correction for covariates that can affect IR (such as age, chemotherapy dosage, GvHD, and steroid-treatment) [9, 64–66] in multivariate analyses for more robust statistical testing.

Our in-depth immune monitoring after omidubicel transplantation also shows robust reconstitution of a broad range of immune cell subsets of CD4+ and CD8+ T-cells, Tregs, gamma-delta T-cells, as well as monocytes, conventional and plasmacytoid DCs. The potent recovery of in particular CD4+ T-cells (>50 × 10^6^ CD4+ T-cells/L blood within 100 days), might be of interest for outcome after omidubicel transplantation since recent evidence suggests that adequate CD4+ IR is related to lower morbidity and mortality after HCT [1–3, 7, 53]. Firm conclusions on CD4+ IR potency, and its’ effect on the outcome, as well as on the broadness of IR in omidubicel recipients are limited by the small number of the evaluated cohort. Nevertheless, these findings indicate that nicotinamide exposure seems to preserve the high IR-potential of CB-grafts, and the ability of the stem and progenitor cells to reconstitute the full range of immune cell subsets in the periphery.

Plasma proteins are currently in the picture as potential biomarkers for outcome after HCT. For instance, early protein profiles of ST2, REG3α, TNFR1, and IL-2Rα were recently reported as predictors for aGvHD severity [67, 68]. In our report, we are the first to show that plasma protein profiles in the first week after omidubicel transplantation correlates with IR data in the weeks thereafter. In particular, we found indications that increased early IL15 plasma concentrations can be related to the fast NK-cell recovery after omidubicel transplantation. IL15 is known to activate NK-cells and improve their function [69, 70]. In addition, we found that IL22 correlates to B-cell reconstitution, while IL22 (produced by immune cells and mucosal epithelial cells) has been linked to B-cell function [71, 72]. Interestingly, we observed that some plasma proteins, such as ST2, are positively related to adaptive immune cell recovery, but negatively to innate immune cell subsets. The role of the ST2 pathway in both adaptive and innate immune cell function is reviewed elsewhere [73]. In turn, higher concentrations of proteins as CD40L, APRIL, and M-CSF, are related to increased innate cell counts and decreased adaptive immune cell counts. These proteins have all been linked to innate as well as adaptive immunity [74–77]. Nevertheless, due to the sparseness of the datasets for these multilayer analyses, we cannot draw strong conclusions from these observations. For this, these relations between plasma proteins and immune subset recovery should be evaluated in larger prospective cohorts, including more patients and more HCT-graft types. In addition, while we focused on the relationship between plasma protein profiles and IR, it is of high interest to further relate this to outcome in a future study with more patients and data.

This study shows that omidubicel transplantation in adolescent and adult patients results in fast and diverse IR that is comparable to reference cohorts of conventional unCBT and BMT at the least. Future studies are needed to further evaluate how IR relates to the outcome, and especially if the enhanced NK-cell and B-cell IR after omidubicel transplantation result in favorable outcomes for patients with hematopoietic malignancies. The results of the present study show that omidubicel transplantation is a potent alternative cell source for HCT in adolescent and adult patients.

## Supplementary information

Supplemental data
