# Supplementary material for: Lymphoid and myeloid immune cell reconstitution after nicotinamide-expanded cord blood transplantation
Source: Bone Marrow Transplant. 2021 Jul 26;56(11):2826–33. doi: 10.1038/s41409-021-01417-4 (PMC8563413; doi:10.1038/s41409-021-01417-4)
Supplement: Supplementary file 1 — Supplemental data [file 41409_2021_1417_MOESM1_ESM.pdf]

Supplementary

**Supplemental Table 1: monoclonals overview**			
marker	*label*	*vendor*	*cat. no.*
CCR4	BV605	BD Horizon	562906
CCR6	PE	BD Pharmingen	559562
CCR7	APC	R&D Systems	FAB197A-100
CCR10	APC	R&D Systems	FAB3478A
CD14	APC-H7	BD Pharmingen	560180
CD16	V500	BD Horizon	561394
CD19	APC-AF750	Beckman Coulter	A94681
CD19	PerCP-Cy5.5	BD	332780
CD25	PE	BD	341011
CD27	APC	BD	337169
CD27	APC-eFluor780	eBioscience	47-0279-42
CD24	PE-CF594	BD	562405
CD3	AF700	BioLegend	300424
CD303	FITC	Miltenyi	130-090-510
CD303	PE-Cy7	BioLegend	354214
CD38	PerCP-Cy5.5	BD Pharmingen	551400
CD4	PerCP-Cy5.5	BD	332772
CD45	Pacific Blue	Dako	PB98601
CD45RA	BV711	BioLegend	304137
CD45RO	BV711	BioLegend	304236
CD56	PE-Cy7	BD	335826
CD56	AF700	BD	557919
CD8	PE-Cy7	BD	335822
CD127	BV421	BD Horizon	562436
CXCR3	FITC	BioLegend	353704
FoxP3	APC	eBioscience	17-4776-42
HLA-DR	Pacific Blue	BioLegend	307633
IgA	PE	Southern Biotech	2052-09
IgD	BV510	BioLegend	348220
IgG	FITC	Southern Biotech	2042-02
IgM	BV421	BD	562618
TCRgd	PE	BD	333141
TCRVα24	PE	Beckman Coulter	IM2283
TCRVβ11	FITC	Beckman Coulter	IM1586
CD123	FITC	Life Tech	11-1239-42
CD11c	PE-CF594	BD	562393

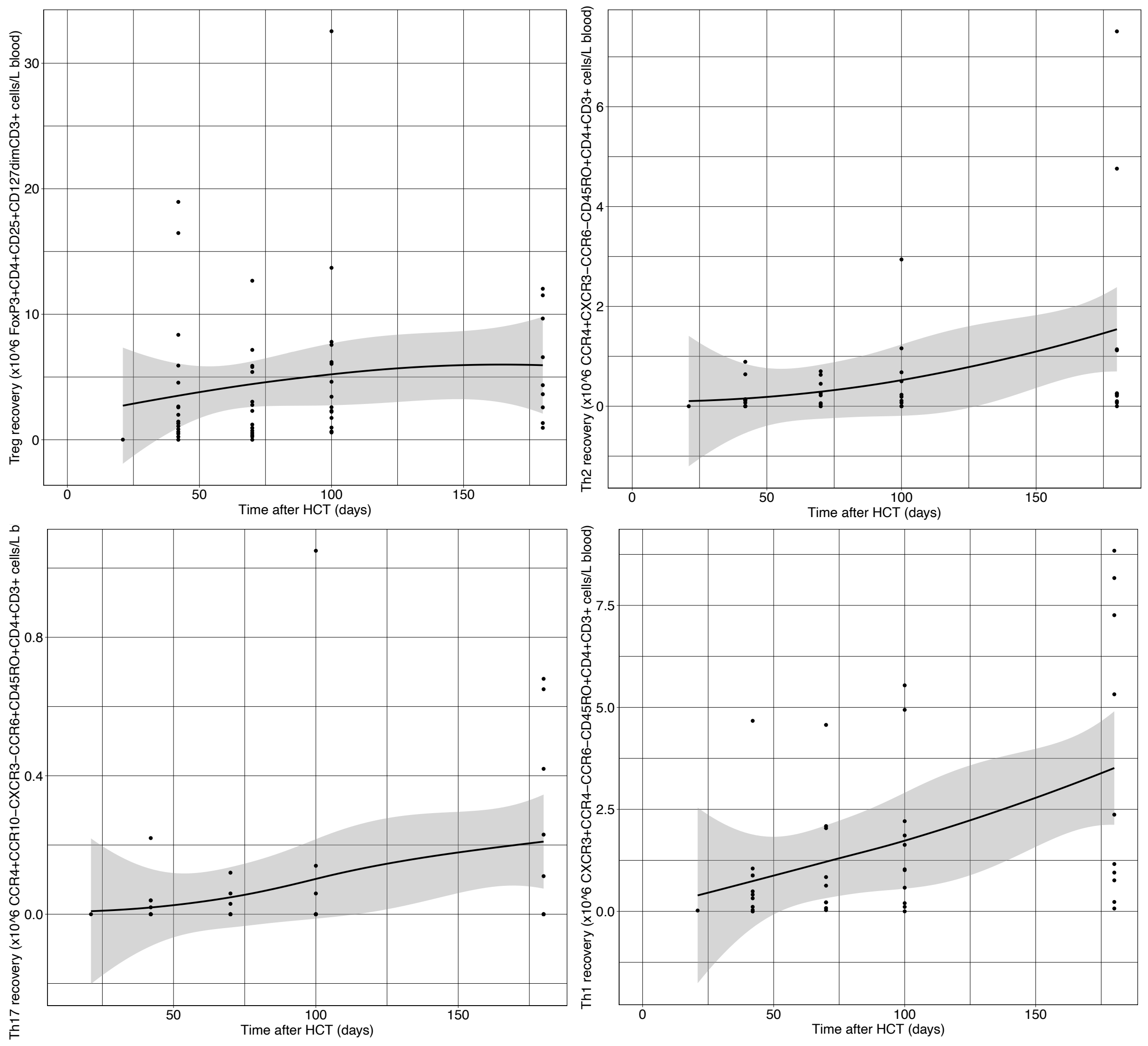

Supplemental Figure 1: CD4+ T-cell subset reconstitution after omidubicel transplantation. Smoothened LOESS-curve with 95% confidence interval (grey area), with dots showing the data points, for absolute CD4+ T-cell subset counts following omidubicel transplantation. Each dot represents a single datapoint for a single patient, showing the data points, of Tregs, Th2, Th1, and Th17 cells.

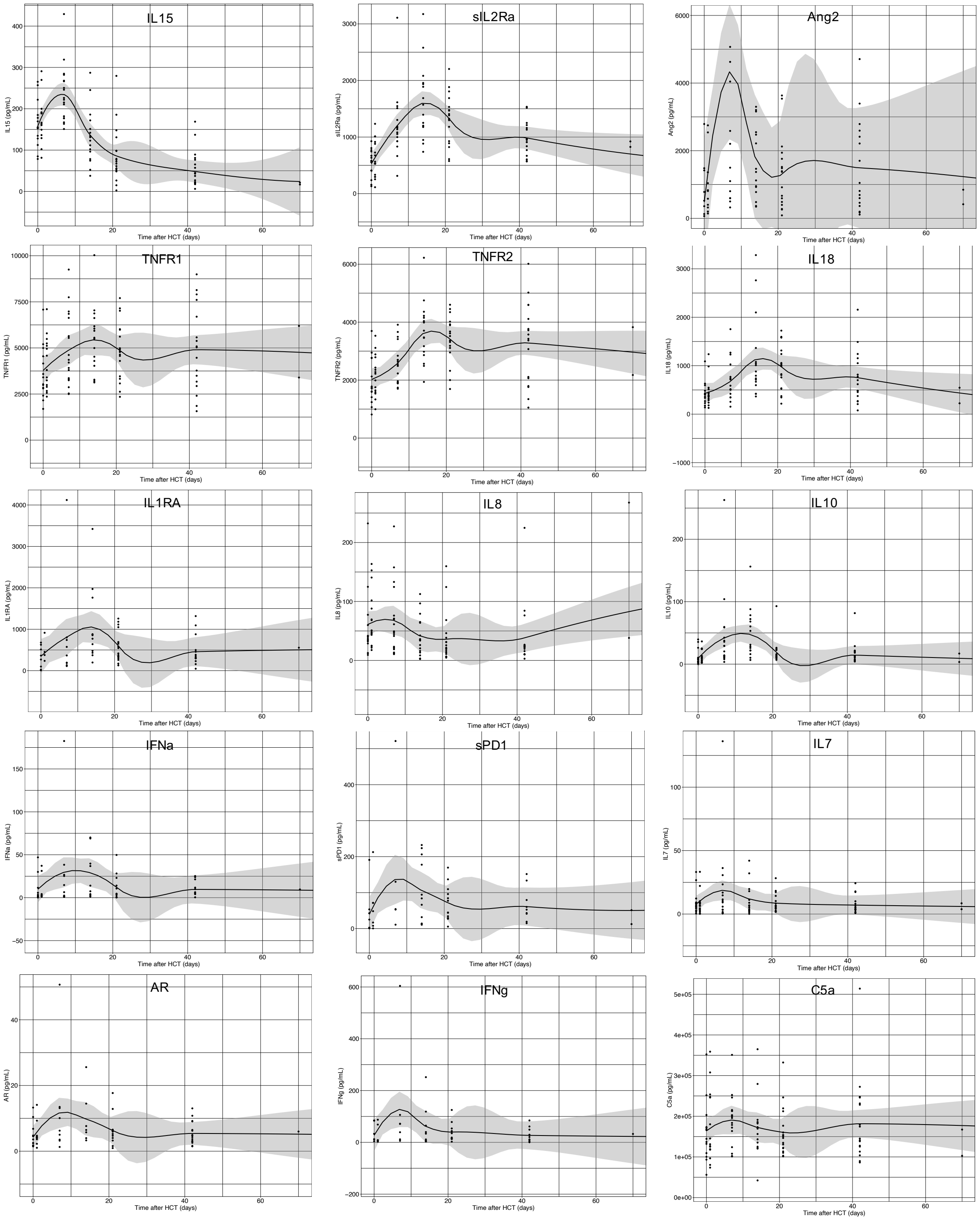

Supplemental Figure 2.1: Plasma protein profiles after omidubicel transplantation. Smoothened LOESS-curve with 95% confidence interval (grey area), with dots showing the data points. Each dot represents a single data point for a single patient, at 0, 1, 7, 14, 21, 42, and 70 days after transplantation. Curves are sorted based on comparable profile types.

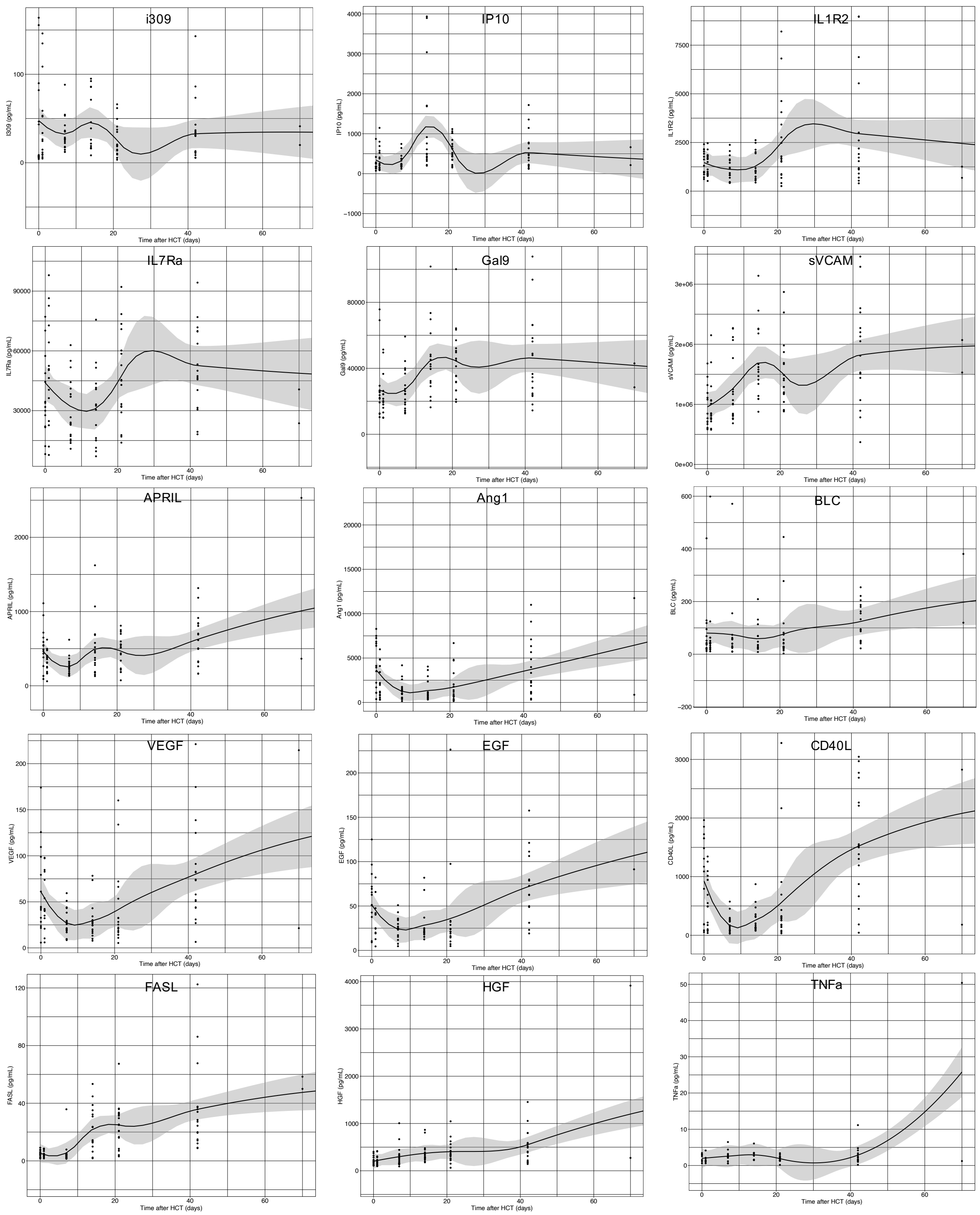

Supplemental Figure 2.2: Plasma protein profiles after omidubicel transplantation. Smoothened LOESS-curve with 95% confidence interval (grey area), with dots showing the data points. Each dot represents a single data point for a single patient, at 0, 1, 7, 14, 21, 42, and 70 days after transplantation. Curves are sorted based on comparable profile types.

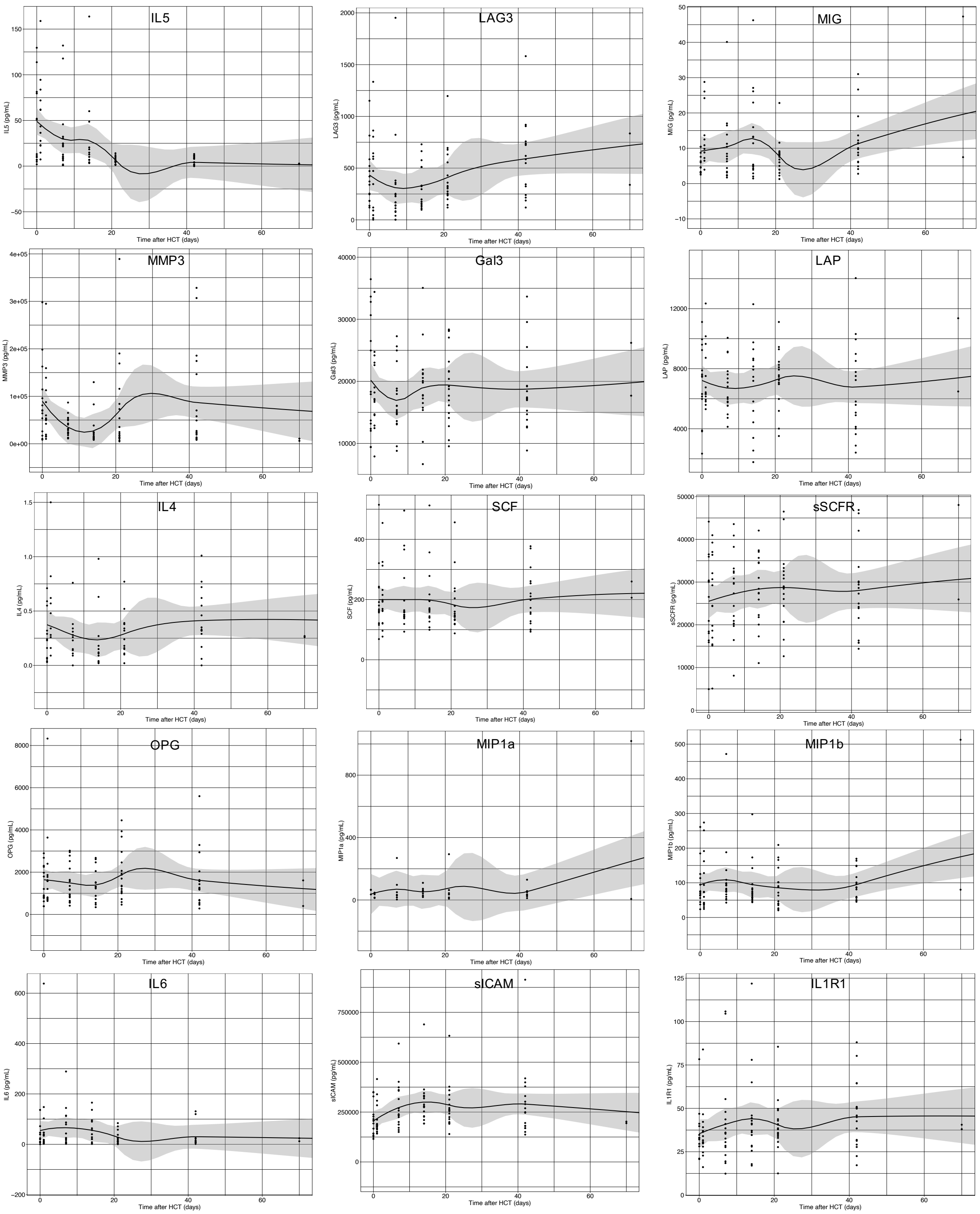

Supplemental Figure 2.3: Plasma protein profiles after omidubicel transplantation. Smoothened LOESS-curve with 95% confidence interval (grey area), with dots showing the data points. Each dot represents a single data point for a single patient, at 0, 1, 7, 14, 21, 42, and 70 days after transplantation. Curves are sorted based on comparable profile types.

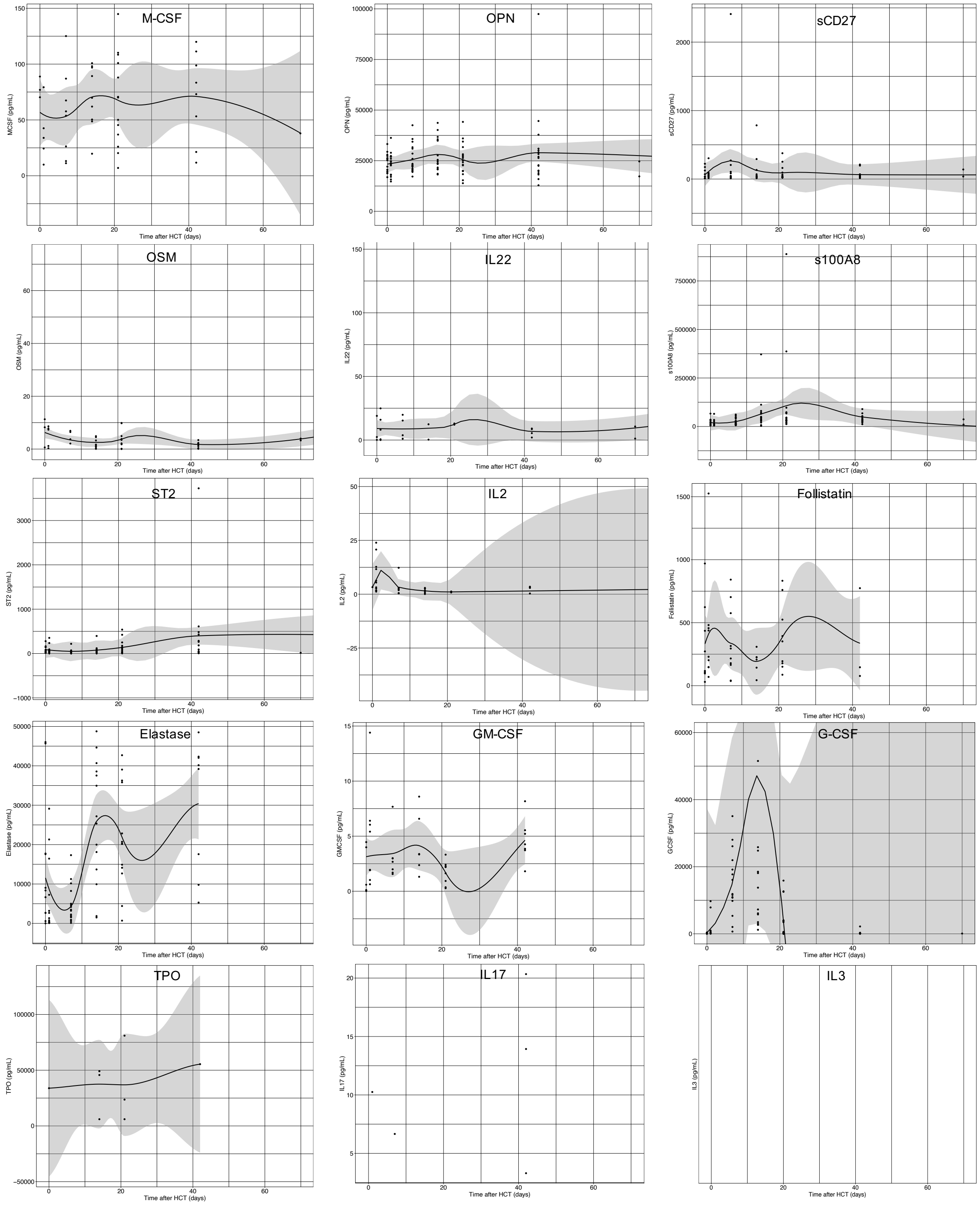

Supplemental Figure 2.4: Plasma protein profiles after omidubicel transplantation. Smoothened LOESS-curve with 95% confidence interval (grey area), with dots showing the data points. Each dot represents a single data point for a single patient, at 0, 1, 7, 14, 21, 42, and 70 days after transplantation. Curves are sorted based on comparable profile types.
